# Supplementary material for: The human brain connectome weighted by the myelin content and total intra-axonal cross-sectional area of white matter tracts
Source: Netw Neurosci. 2023 Dec 22;7(4):1363–88. doi: 10.1162/netn_a_00330 (PMC10697181; doi:10.1162/netn_a_00330)
Supplement: Supplementary file 1 [file netn-7-4-1363-s001.pdf]

Nelson, M.C., Royer, J., Lu, W.D., Leppert, I.R., Campbell, J., Schiavi, S., Jin, H., Tavakol, S., Vos de Wael, R., Cruces, R.R., Pike, G.B., Bernhardt, B., Daducci, A., Misic, B., & Tardif, C. (2023). The Human Brain Connectome Weighted by the Myelin Content and Total Intra-Axonal Cross-Sectional Area of White Matter Tracts. *Network Neuroscience*, Advance publication. https://doi.org/10.1162/netn_a_00330.

SUPPLEMETARY INFORMATION

The Human Brain Connectome Weighted by the Myelin Content and Total Intra-Axonal Cross-Sectional Area of White Matter Tracts

Mark C. Nelson[1,2], Jessica Royer[1,2], Wen Da Lu[2,3], Ilana R. Leppert[2], Jennifer S.W. Campbell[2], Simona Schiavi[4], Hyerang Jin[1,2], Shahin Tavakol[1,2], Reinder Vos de Wael[1,2], Raul Rodriguez-Cruces[1,2], G. Bruce Pike[5], Boris C. Bernhardt[1,2], Alessandro Daducci[4], Bratislav Misic[1,2], and Christine L. Tardif [1,2,3]

[1]Department of Neurology and Neurosurgery, McGill university, Montreal, QC, Canada. [2]McConnell Brain Imaging Centre, Montreal Neurological Institute and Hospital, Montreal, QC, Canada. [3]Department of Biomedical Engineering, McGill University, Montreal, QC, Canada. [4]Department of Computer Science, University of Verona, Verona, Italy. [5]Hotchkiss Brain Institute and Departments of Radiology and Clinical Neuroscience, University of Calgary, Calgary, Canada.

Corresponding Author: Mark C Nelson, mark.nelson3@mail.mcgill.ca

Robustness of Results across Parcellation Resolutions

We replicated all primary analyses across a wide range of resolutions of the Schaefer cortical parcellation. When correlating edge-length-regressed residuals with FC across the 200-, 400- and 600-node Schaefer atlas (**Figure S1A**), the negative correlation with R_1 and positive correlation with NoS, SIFT2 and COMMIT are all robust. Correlation magnitude tends to increase with

parcellation granularity. This trend is reversed in the very-high resolution Schaefer-900 (**Figure S1B**).

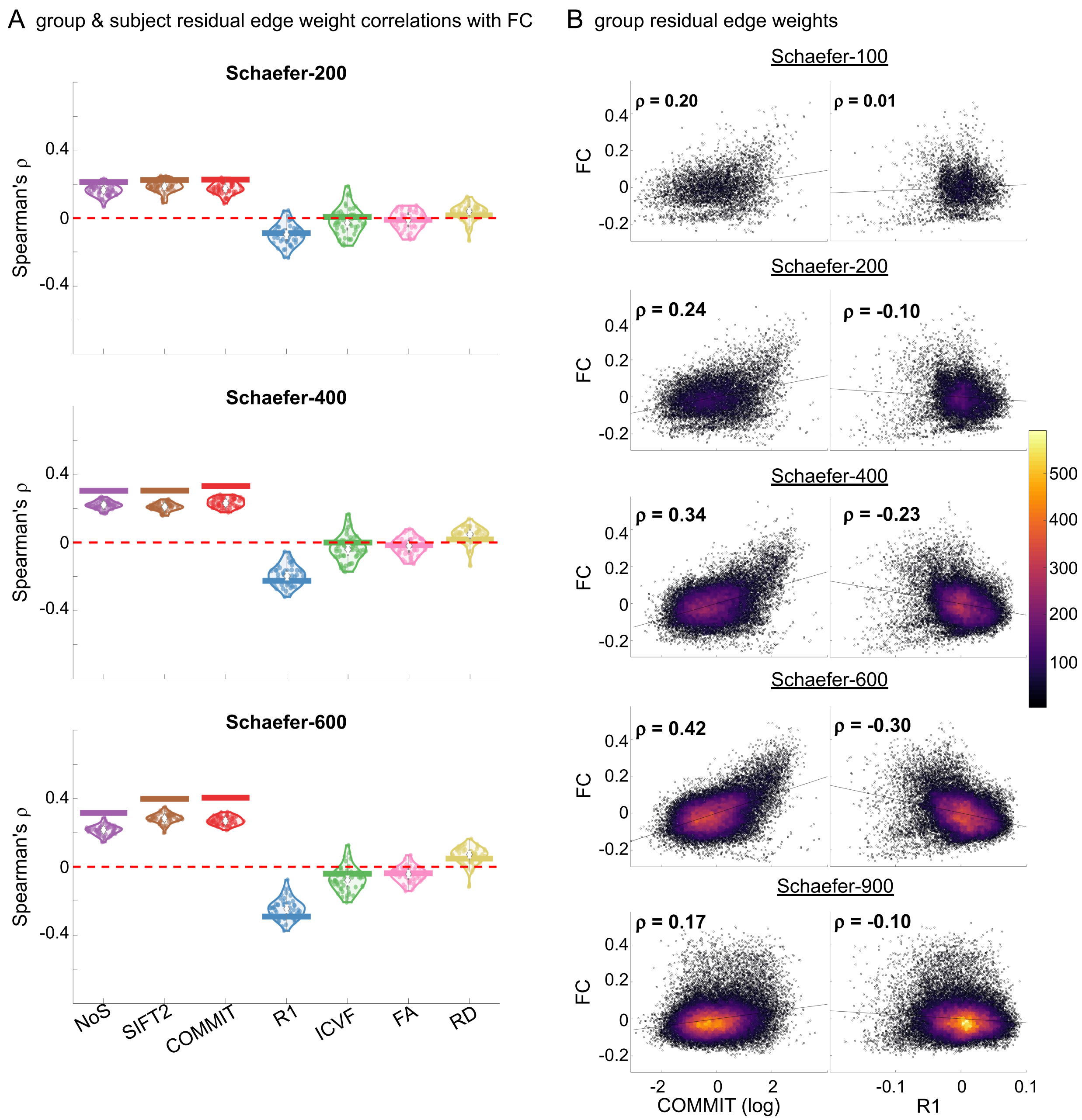

Figure S1. *Residual Edge Weight Correlations with FC across Parcellation Resolution. (A) Violin distributions of edgewise Spearman's rank correlations of residual edge weights in all networks with residual edge weights in FC. Nodes are defined by the 200, 400 and 600 cortical node Schaefer*

parcellation. Residual edge weights were computed by linear regression of edge length. Colored data points and bars respectively indicate subject-level and group-level correlations. (B) Heat scatter plots of group-level residual edge weights from FC as a function of COMMIT (left) and R_1 (right) across the 100, 200, 400, 600 and 900 node Schaefer cortical parcellation. Spearman's rank correlations are reported (ρ), and the best fit linear curve is shown in black. Color indicates data density. All networks additionally include 14 subcortical regions.

When correlating edge-length-regressed residuals with R_1 across the 100, 200, 400,600 and 900 node Schaefer atlas (**Figure S2A**), the negative correlation with NoS, SIFT2, COMMIT and ICVF are all robust. Interestingly, while correlation magnitude tends to increase with parcellation granularity in the streamline-specific networks, it follows the reverse trend for ICVF. Scatter plots for these data are also shown (**Figure S2B**). The inverse relationship between R_1 and COMMIT is found to be driven by the shortest network edges in the Schaefer-200 (**Figure S2C**) similarly to the result reported in the Schaefer-400.

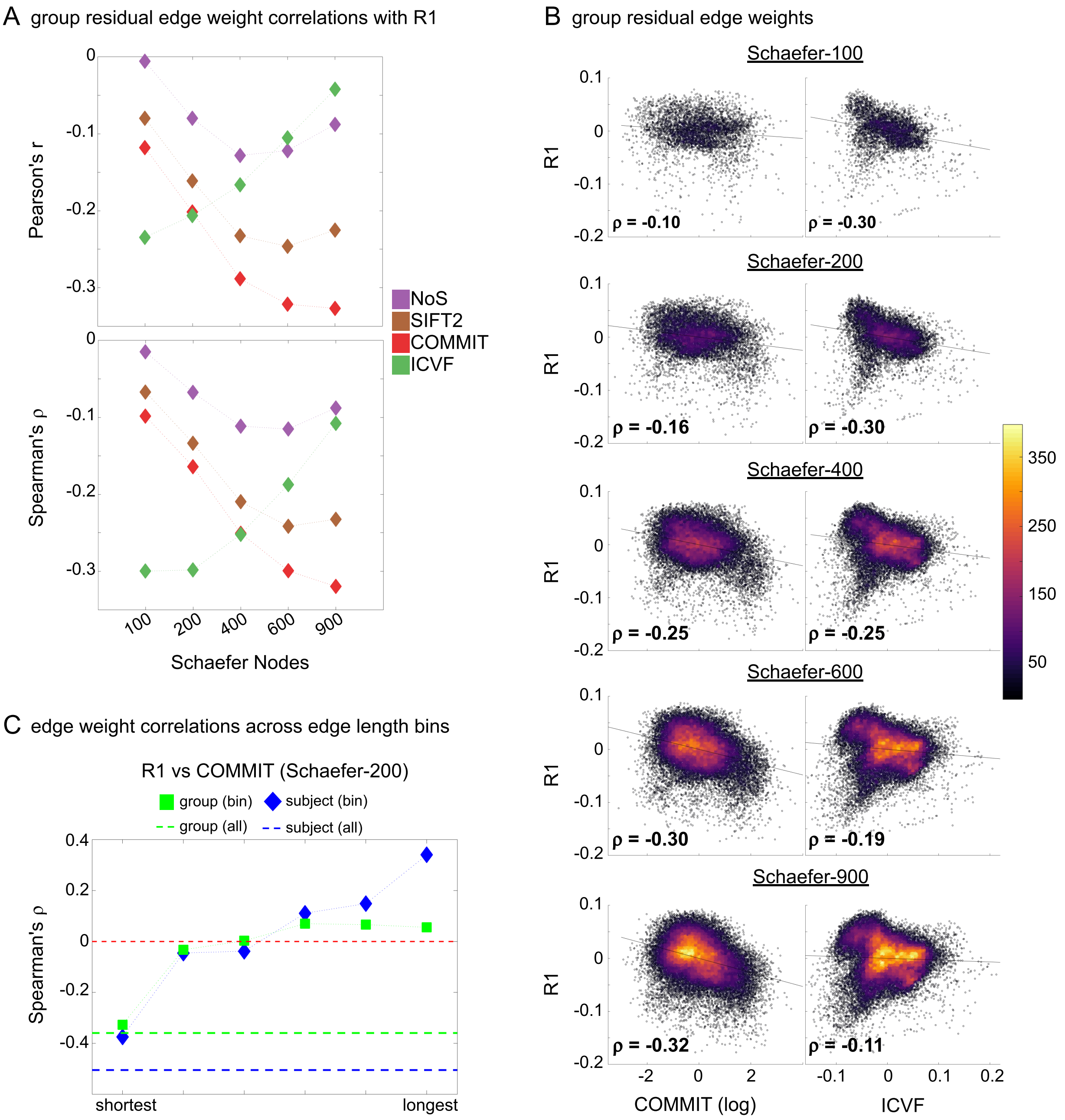

Figure S2. *Residual Edge Weight Correlations with R_1 across Parcellation Resolution. (A) Group-level edgewise Pearson's r (top) and Spearman's ρ (bottom) of edge-length-regressed-residual edge weights from R_1 with NoS, SIFT2, COMMIT and ICVF across the 100, 200, 400, 600 and 900 node Schaefer parcellation. (B) Heat scatter plots of group-level residual edge weights from R_1 as a function of COMMIT (left) and ICVF (right) across the 100, 200, 400, 600 and 900 node Schaefer parcellation. Spearman's rank correlations are reported (ρ), and the best fit linear curve is shown in black. Color*

indicates data density. (C) Edgewise Spearman's rank correlation of edge weights in R_1 vs COMMIT across edge length bins for the Schaefer-200. Group-level and subject-level are respectively shown in green and blue. The square and diamond markers connected by dotted lines show binned correlation values, and the horizontal dashed green and blue lines mark the correlation values for all edges pooled together. All networks additionally include 14 subcortical regions.

When replicating the graph theory analysis for group-level networks in the Schaefer-200 and -600 parcellation (**Figure S3**), all weighted structural networks display normalized small-worldness. Once again, this feature is higher in magnitude for COMMIT, SIFT2 and NoS relative to the tractometry networks, which are essentially indiscernible. Small-worldness increases with parcellation granularity in all structural networks. A weighted rich club is detected in COMMIT, SIFT2 and NoS, as well as the binary network over a large range of degree k. As in the main text, FA and R_1 do not show a weighted rich-club topology for any degree k.

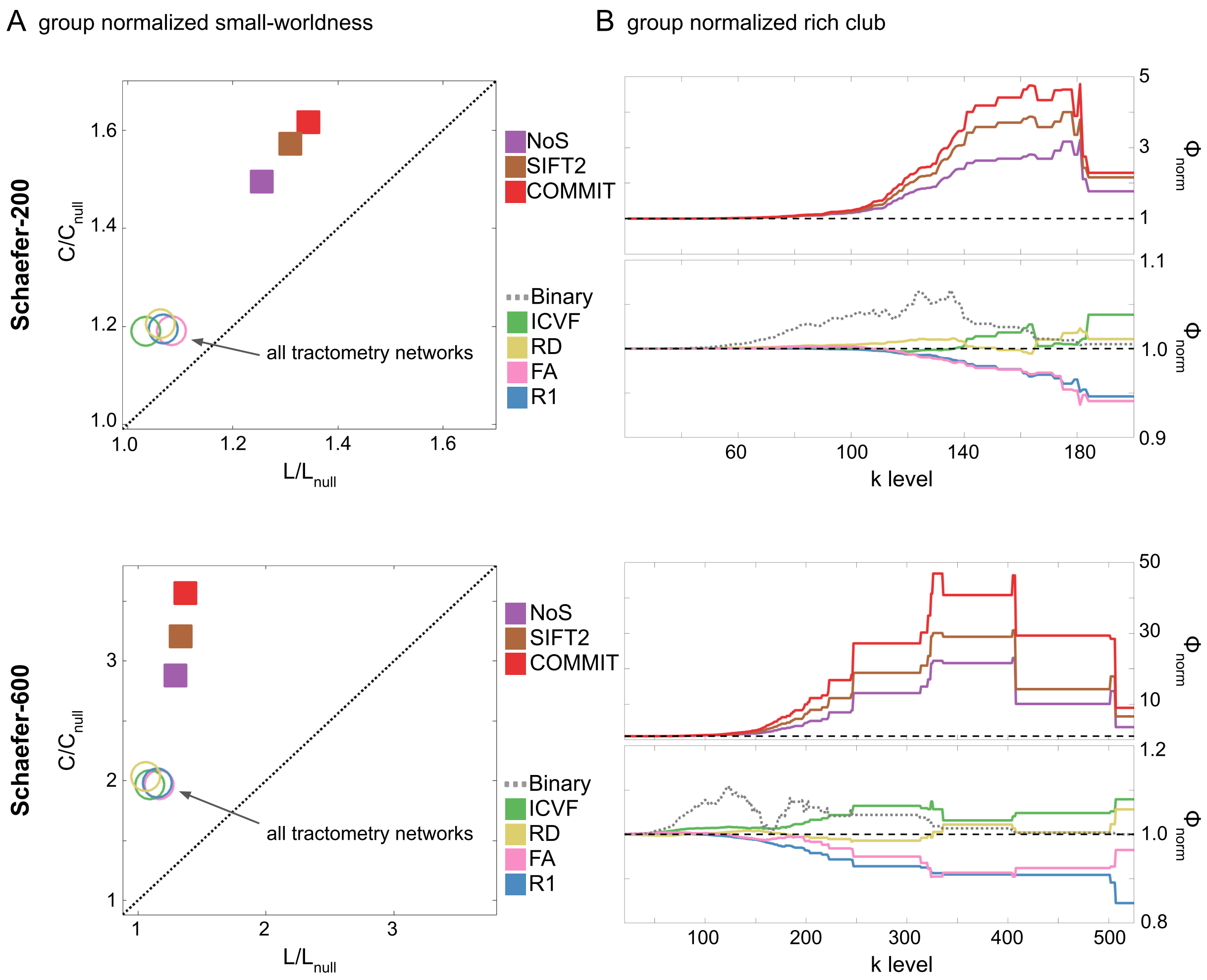

Figure S3. *Group-Level Network Topology across Parcellation Resolution. (A) Small-worldness was estimated in all structural networks: clustering coefficient was normalized within each node, averaged across nodes (C/C_{null}), then plot as a function of normalized characteristic path length (L/L_{null}). Topology measures averaged across 50 degree and strength preserving null networks were used for normalization. Networks above the identity line (dotted black) are characterized by the small world attribute. Tractometry networks are indicated by the arrow. (B) Normalized rich club curves are shown for COMMIT, NoS and SIFT2 (top), as well as ICVF, RD, FA and R_1 (bottom). A single binary network (dotted gray line) is also shown (bottom) as binary connectivity was uniform across weighted networks. The normalized rich-club coefficient (ϕ_{norm}) was computed across the range of degree (k) and normalized against 1000 null networks (degree preserving for binary and degree and strength preserving for*

weighted networks). A ϕ_{norm} value > 1 (horizontal dashed black lines) over a range of k indicates the presence of a rich club. Analyses were replicated in the 200 (top) and 600 (bottom) node Schaefer parcellation. All networks additionally include 14 subcortical regions.

Normalized hubness (**Figure S4**) is observed to change for each weighted structural network across the 200, 400 and 600 node Schaefer parcellations. We suspect that it may be due in part to the inclusion of 14 subcortical ROIs, which remain static in granularity. Thus, the subcortical nodes become increasingly more densely connected with respect to the cortex as the granularity of the cortical parcellation is increased.

Group Normalized Hubness

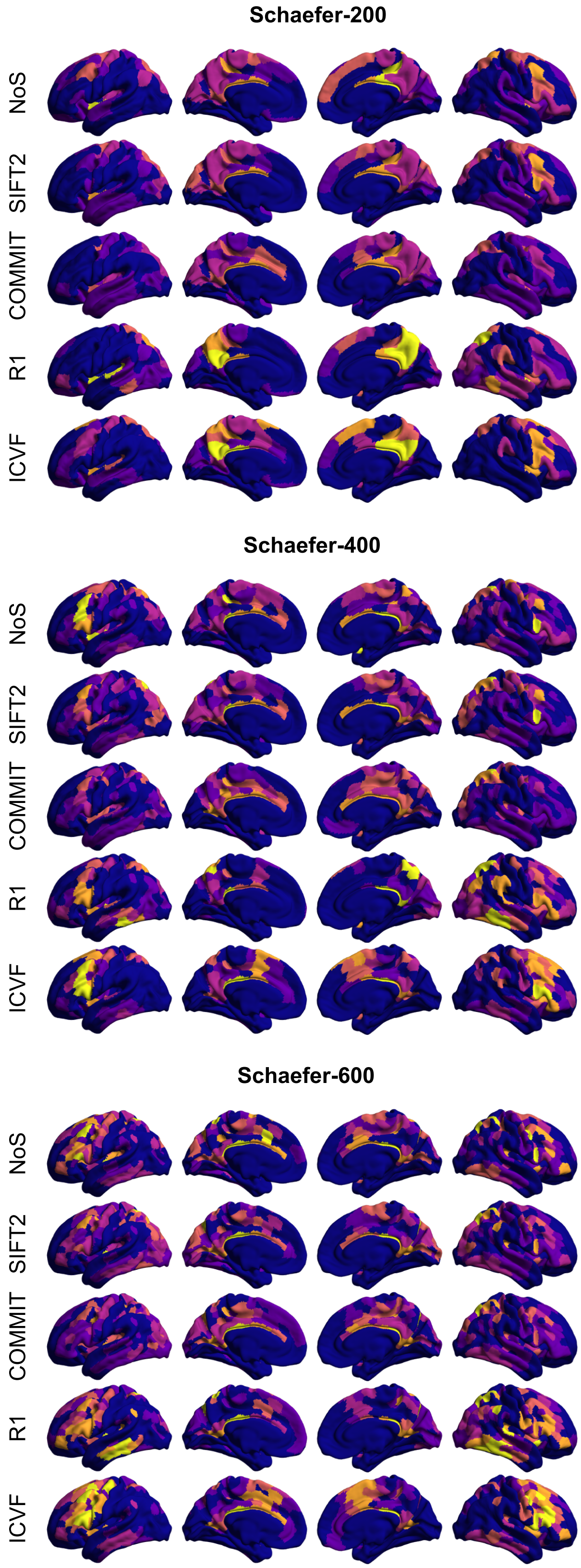

***Figure S4**. Group-Level Normalized Hubness across Parcellation Resolution. Nodewise hubness scores are projected onto the 200, 400 and 600 node Schaefer cortical parcellation plus 14 subcortical regions (not shown). Scores (0-5) were computed for each node as +1 point for all nodes in top 20% strength, betweenness, closeness and eigenvector centrality, as well as bottom 20% clustering coefficient.*

Replication in a Second Dataset

All primary analyses were successfully replicated (**Figure S5**-**S6**) in a second multimodal MRI dataset acquired at a lower resolution and comprising 20 healthy volunteers scanned at 3 Tesla on a Siemens Magnetom Prisma-Fit scanner equipped with a 64-channel head coil including:

- Multi-shell diffusion-weighted imaging (DWI): 2D pulsed gradient spin-echo echo-planar imaging sequence consisting of shells with b-values 0, 300, 1000, and 2000s/mm^2 in 6, 10, 30, and 64 diffusion directions, respectively (2.6mm isotropic; TR = 3000ms, TE = 57ms; multi-band factor = 3). b0 images were also acquired with reverse phase encoding direction to facilitate distortion correction of DWI data.
- Quantitative T_1 relaxometry data was acquired with a sparse 3D-MP2RAGE sequence (Marques et al., 2010) (1mm isotropic; TR = 5000ms, TE = 2.7ms, TI_1 = 940ms, $T1_2$ = 2830ms; partial Fourier = 6/8). This was used to compute a T_1 map which was sampled to estimate the edge weights in networks weighted by R_1 (myelin-weighted).

Preprocessing deviated in two important ways: (1) tractograms of only 1M streamlines were used (vs 5M in the main text), and (2) the edge weights in NoS-weighted networks were not scaled by the inverse of node volume. All other methods were identical.

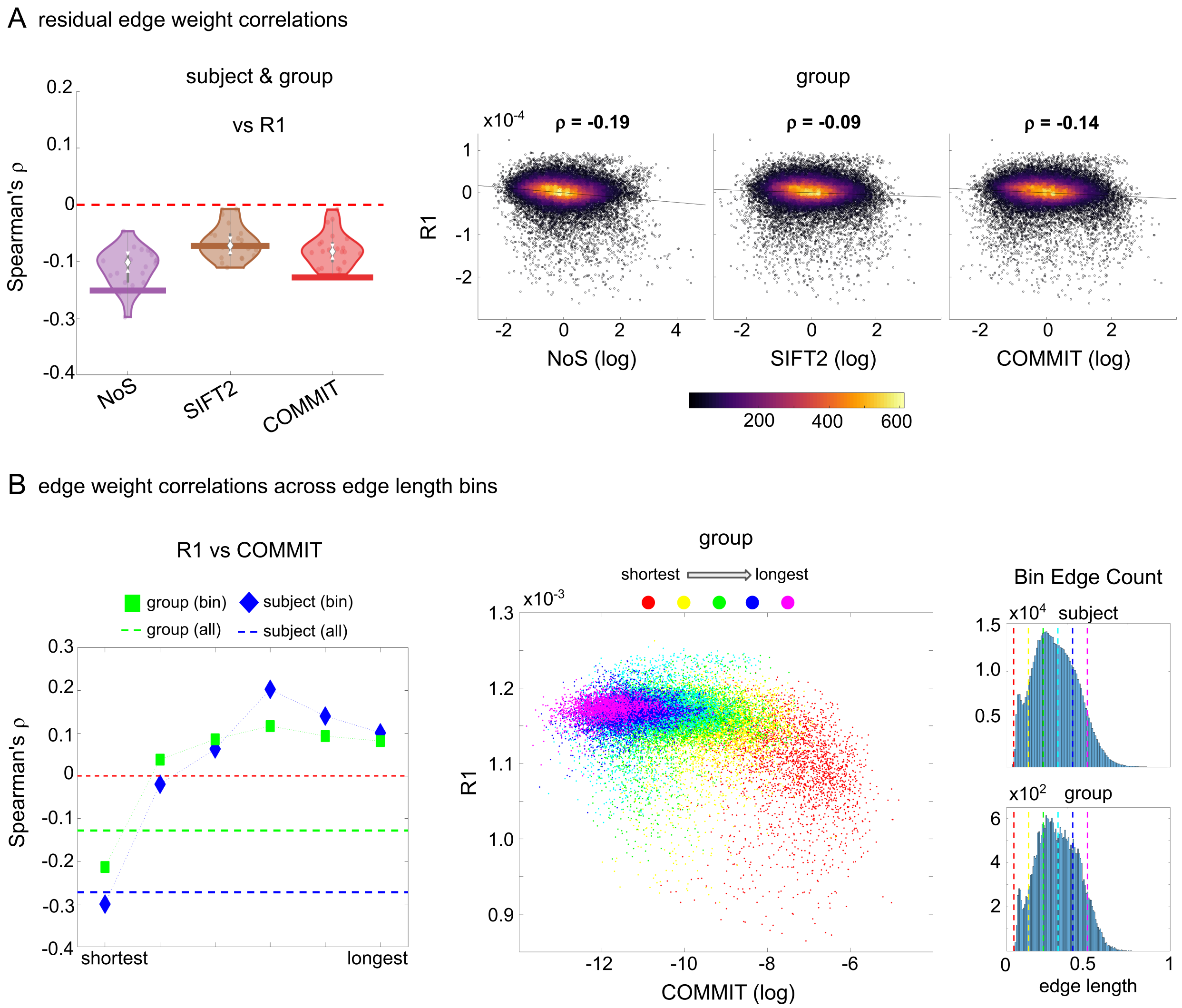

Figure S5. *The Myelin-Dependence of Structural Brain Networks Replicated. (A) Violin distributions (left) of edgewise Spearman's rank correlations with the myelin-weighted network R_1. Residual edge weights are compared following linear regression of edge length. Colored data points and bars respectively indicate subject-level and group-level correlations. Heat scatter plots (right) of group-level residual edge weights in R_1 as a function of NoS, SIFT2 and COMMIT. Spearman's rank correlations are reported (ρ), and the best fit linear curve is shown in black. Color indicates data density. (B) Line plot (left) of edgewise Spearman's rank correlation of edge weights in R_1 vs COMMIT across edge length bins. Group-level and subject-level are respectively shown in green and blue. The square and diamond*

markers connected by dotted lines show binned correlation values, and the horizontal dashed green and blue lines mark the correlation values for all edges pooled together. Scatter plot (middle) of group-level edge weights in R_1 as a function of COMMIT with data points colored by bin identity. Histograms (right) illustrating subject- and group-level edge length bins.

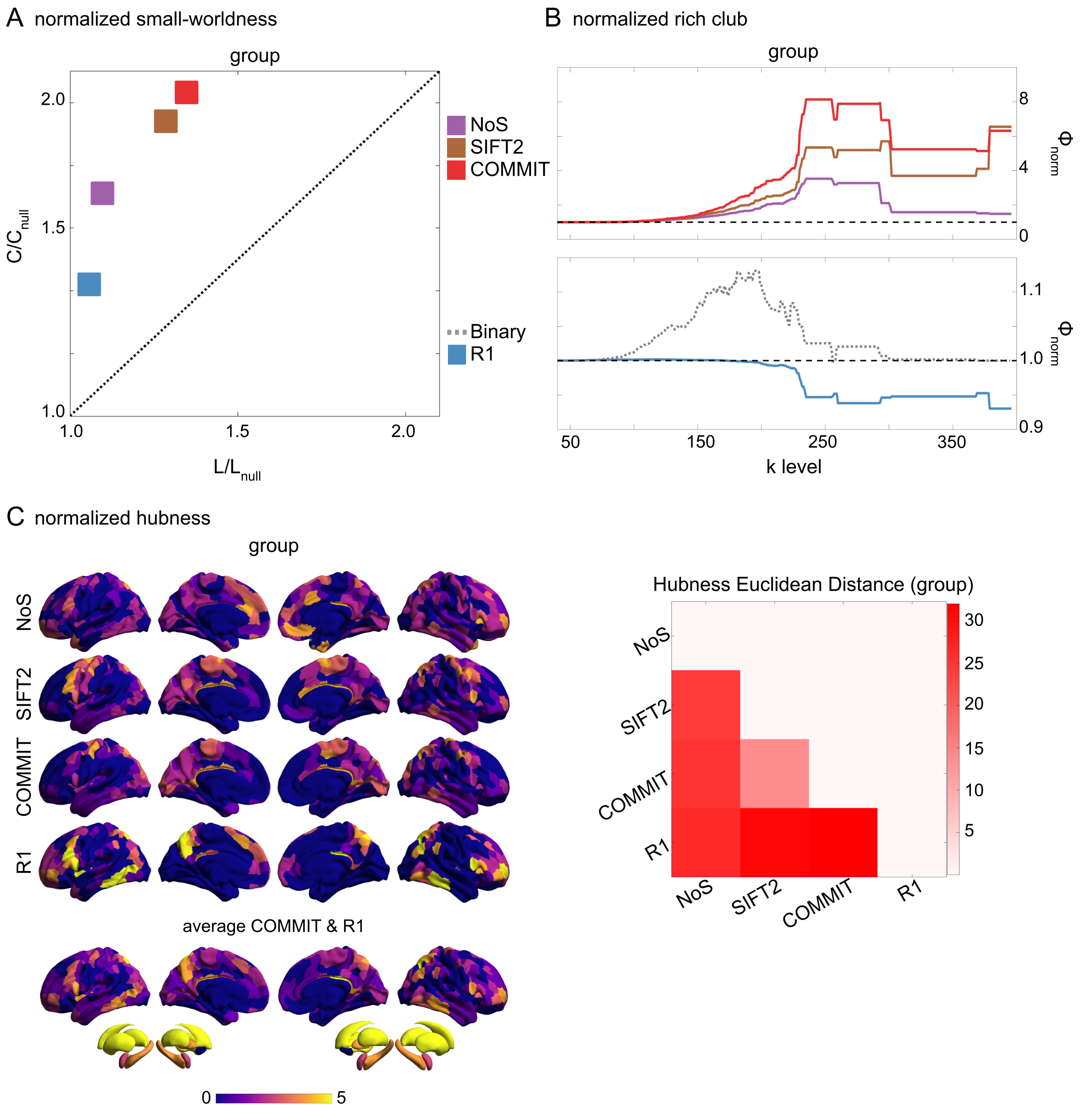

Figure S6. *Group-Level Network Topology Replicated. (A) Small-worldness was estimated in all structural networks: clustering coefficient was normalized within each node, averaged across nodes (C/C_{null}), then plot as a function of normalized characteristic path length (L/L_{null}). Topology measures averaged across 50 degree and strength preserving null networks were used for normalization. Networks above the identity line (dotted black) are characterized by the small world attribute. (B) Normalized rich-club curves are shown for COMMIT, NoS and SIFT2 (top), as well R_1 (bottom). A single binary network*

(dotted gray line) is also shown (bottom) as binary connectivity was uniform across weighted networks. The normalized rich-club coefficient (ϕ_{norm}) was computed across the range of degree (k) and normalized against 1000 null networks (degree preserving for binary and degree and strength preserving for weighted networks). A ϕ_{norm} value > 1 (horizontal dashed black lines) over a range of k indicates the presence of a rich club. (C) Nodewise hubness scores are projected onto Schaefer-400 cortical and 14-ROI subcortical surfaces. Scores (0-5) were computed for each node as +1 point for all nodes in top 20% strength, betweenness, closeness and eigenvector centrality, as well as bottom 20% clustering coefficient. The matrix (right) shows the Euclidean distance between all pairs of nodal hubness vectors.

Permutation Testing

We assessed the significance of edgewise correlations with FC (**Figure S7**) and R_1 (**Figure S8**) by way of permutation testing. First, variance due to edge length was controlled for in all networks using linear regression. Edge weights in NoS, SIFT2 and COMMIT were log transformed to facilitate linear regression of edge length. Then, the residual edge weights in the network being correlated with either FC or R_1 were shuffled, and a Spearman's rank correlation coefficient was computed. This process was repeated 1000 times for each network pair. Significance was quantified with a one-sided p-value (p_{perm}) as the number of shuffled correlation coefficients equal in sign and greater in magnitude than the empirical value scaled by the number of permutation iterations. The correlation coefficients corresponding to all primary reported results with FC and R_1 were significantly greater in magnitude than their corresponding null distributions: all had P_{perm} values $\cong 0$.

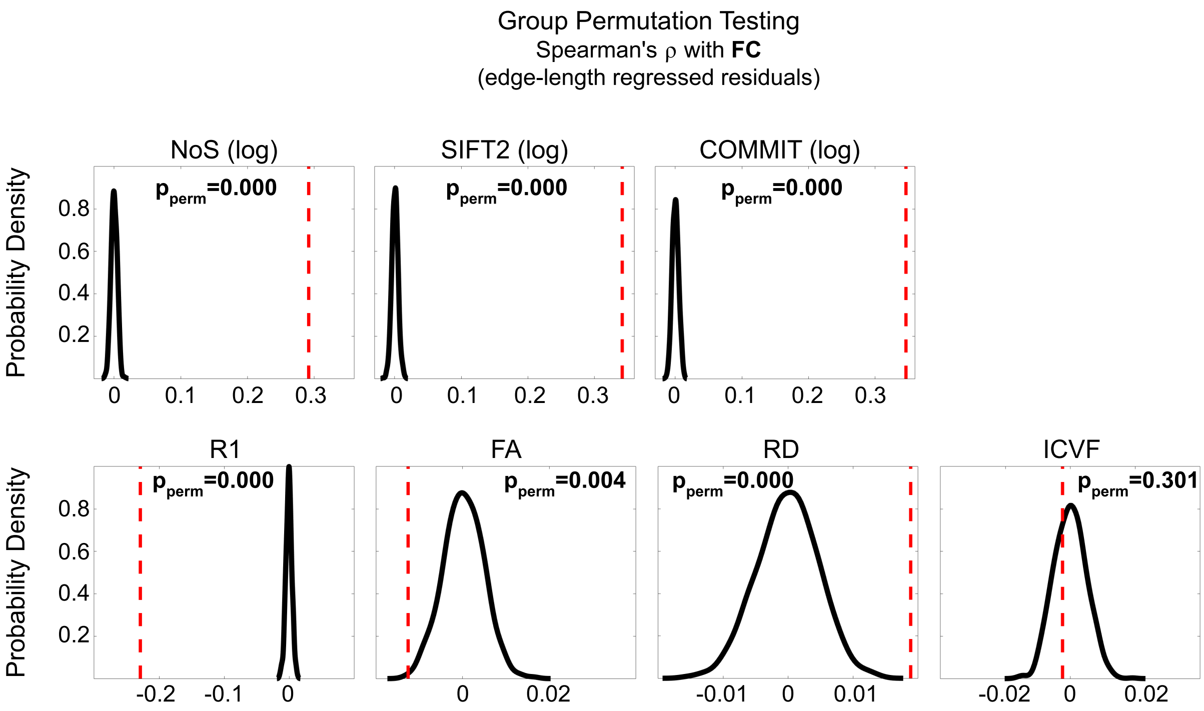

Figure S7*. Permutation Testing of Edgewise Residual Correlations with FC.*

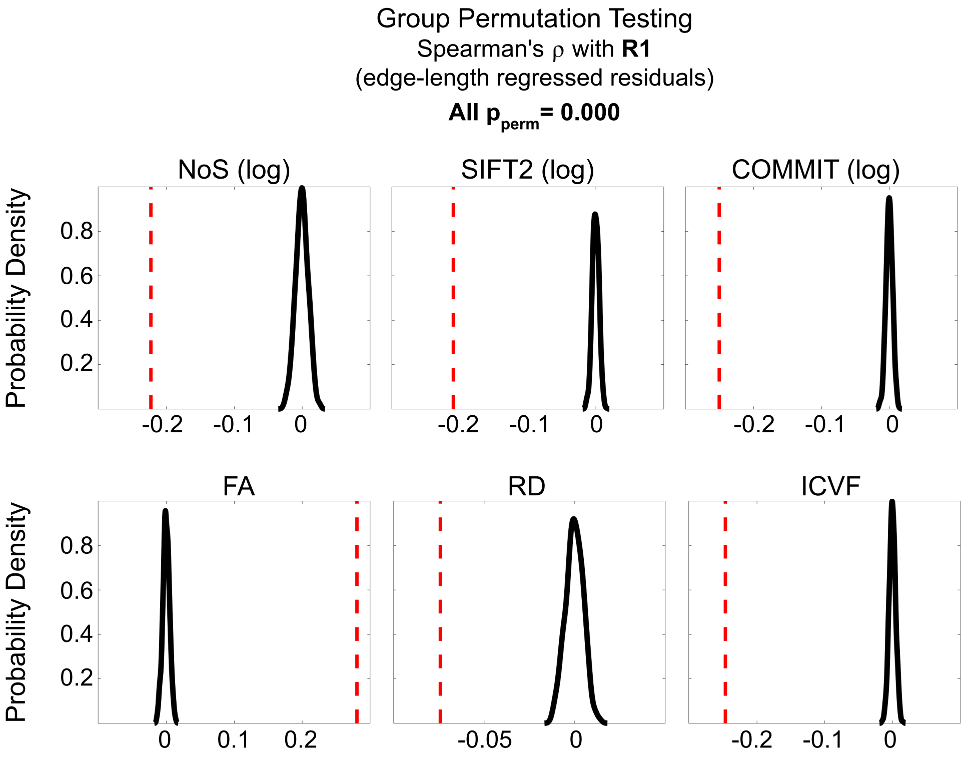

Figure S8*. Permutation Testing of Edgewise Residual Correlations with R_1.*

Intraclass Correlation

To complement the CQD analysis of variance in the main manuscript, we assessed edge weight variance by computing an intraclass correlation (ICC) coefficient within each weighted network in the following manner. First, the edge weights in all networks were normalized to the range [0 1] by dividing by the subject-level maximum value within network. Then, subject-level connectivity matrices were vectorized such that all lower triangle elements excluding the main diagonal were stacked in an edge x subject matrix. Any edge = 0 for all subjects was removed from this matrix. Then, in two variants of this analysis, these edge weights were either (1) not log transformed, or (2) $\log_{10}$ transformed if they were skewed (NoS, SIFT2 and COMMIT). In both

cases, an ICC coefficient was computed using a two-way random effects ANOVA (analysis of variance) model (**Figure S9**).

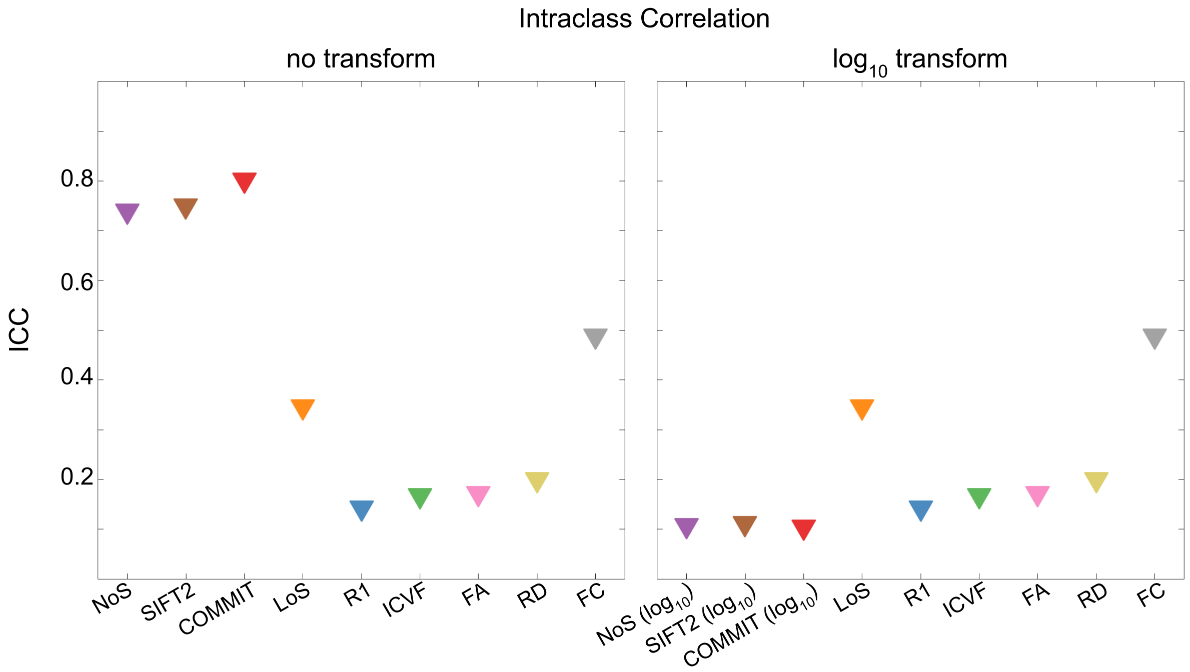

Figure S9*. Intraclass Correlation of Brain Networks.*

When edge weights remained untransformed, ICC was highest for COMMIT (r = 0.80), SIFT2 (r = 0.75) and NoS (r = 0.74). However, log transformation of the edge weights in these networks decreased the magnitude of all of these coefficients to r ≅ 0.10. All tractometry networks had ICC values ranging from 0.14 to 0.20. ICC for LoS and FC was 0.35 and 0.49, respectively. It is important to emphasize that the distribution of edge weights is a fundamental property of a brain network, and a transformation of this property yields a network with transformed features. Given that the ICC is sensitive to the distribution of the input data, in contrast to the CQD which is robust to outliers and skewed data, the comparability of ICC across structural brain networks with fundamentally different edge weight distributions remains unclear.

COMMIT-Weighted Average Tractometry Does Not Alter SC Network Features

In the main text, we have shown features of tractometry-derived SC networks which generally include lower within- and between-subject edge weight contrast, weaker correlations with FC and R_1, as well as lower magnitude small-worldness and rich-club coefficients relative to streamline-specific networks. We have suggested that these characteristics are likely to be due in part to the widespread partial voluming which occurs in the computation of edge weights using the tractometry method. In our main processing pipeline, we have taken measures to minimize the impact of this bias on the edge weights in our tractometry networks by eliminating all streamlines from the tractogram with a zero COMMIT weight (i.e., $< 1e^{-12}$). According to the COMMIT model, these streamlines do not contribute to the global diffusion signal and are thus interpreted as false positives.

The non-zero COMMIT streamline weights could be used to further reduce partial volume effects. Here, we compare in four subjects the tractometry edge weight computation approach described in the main text to a variant that uses the non-zero COMMIT streamline weights to compute a weighted average over streamlines for each node pair. For example, the edge weights in the FA-weighted network derived using the COMMIT-weighted-average method are computed as:

$$\alpha_{ij} = \frac{\sum_{k=1}^{N_{ij}} (x_{ij}^k * FA_{ij}^k)}{\sum_{k=1}^{N_{ij}} (x_{ij}^k)},$$

where α_{ij} is the edge weight between nodes i and j, N_{ij} is the number of streamlines, and FA_{ij}^{k} and x_{ij}^{k} are the FA and COMMIT weights of streamline k. Importantly, streamlines with a COMMIT weight < $1e^{-12}$ are excluded by both methods. Therefore, these results are most accurately interpreted as a comparison of degrees of COMMIT-weighted tractometry.

Using this approach, we report that subject-level edge weight distributions appear qualitatively similar across the original (simple mean across streamlines) and COMMIT-weighted average tractometry methods (**Figure S10**). In addition, patterns of edgewise correlation with FC and R_1 (**Figure S11**), as well as small-worldness and rich-club curves are essentially identical. Thus, at this level of analysis, the COMMIT-weighted average tractometry approach (COMMIT-filtered tractogram → COMMIT-weighted average) does not substantially alter the features of SC networks relative to the tractometry approach (COMMIT-filtered tractogram → simple mean) implemented in the main manuscript.

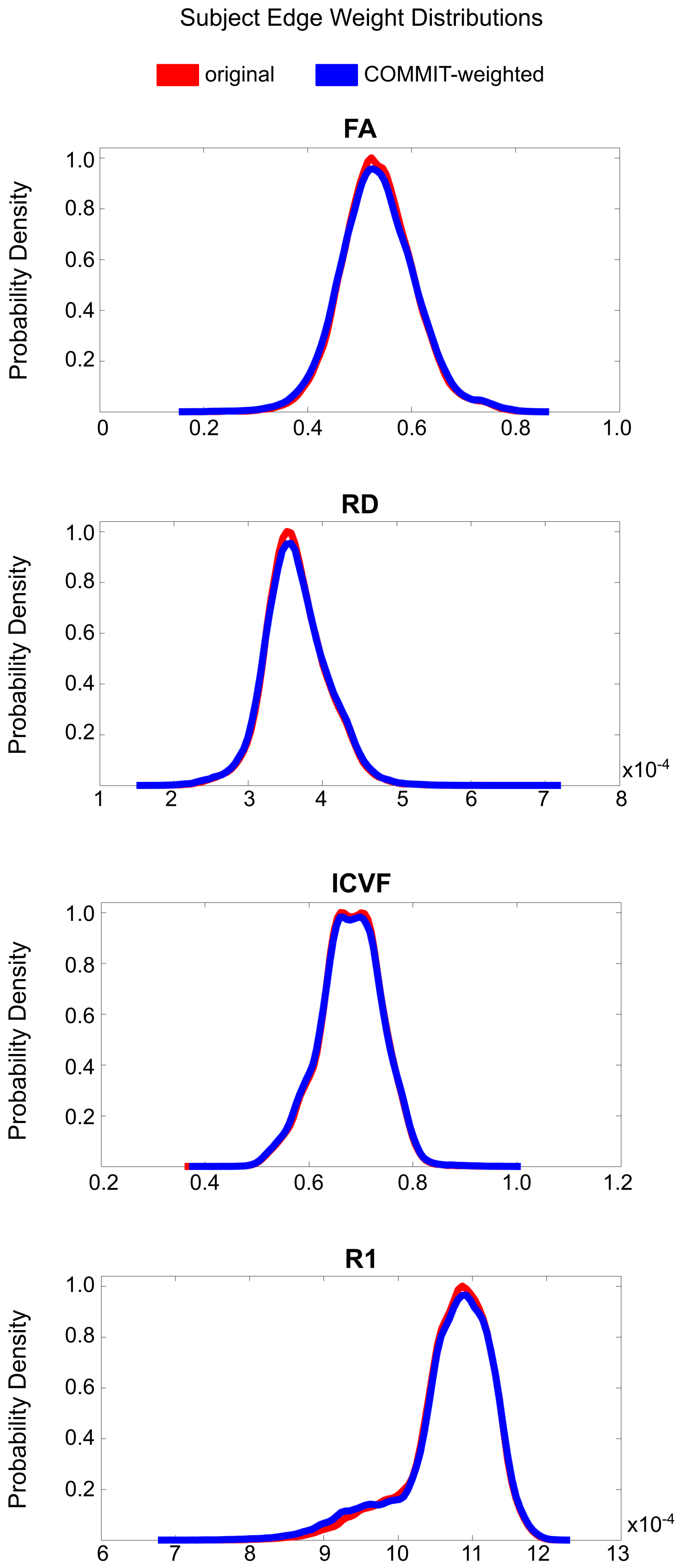

Subject Edge Weight Distributions
original
COMMIT-weighted
FA
Probability Density
1.0
0.8
0.6
0.4
0.2
0
0.2
0.4
0.6
0.8
1.0
RD
Probability Density
1.0
0.8
0.6
0.4
0.2
x10-4
1
2
3
4
5
6
7
8
ICVF
Probability Density
1.0
0.8
0.6
0.4
0.2
0.2
0.4
0.6
0.8
1.0
1.2
R1
Probability Density
1.0
0.8
0.6
0.4
0.2
x10-4
6
7
8
9
10
11
12
13

Figure S10. *Pooled Subject-Level Edge Weight Distributions. Original (simple mean; red) and COMMIT-weighted (weighted average; blue) methods of tractometry edge weight computation are compared across SC networks weighted by FA, ICVF, RD and* R_1. *Data from only four subjects is shown.*

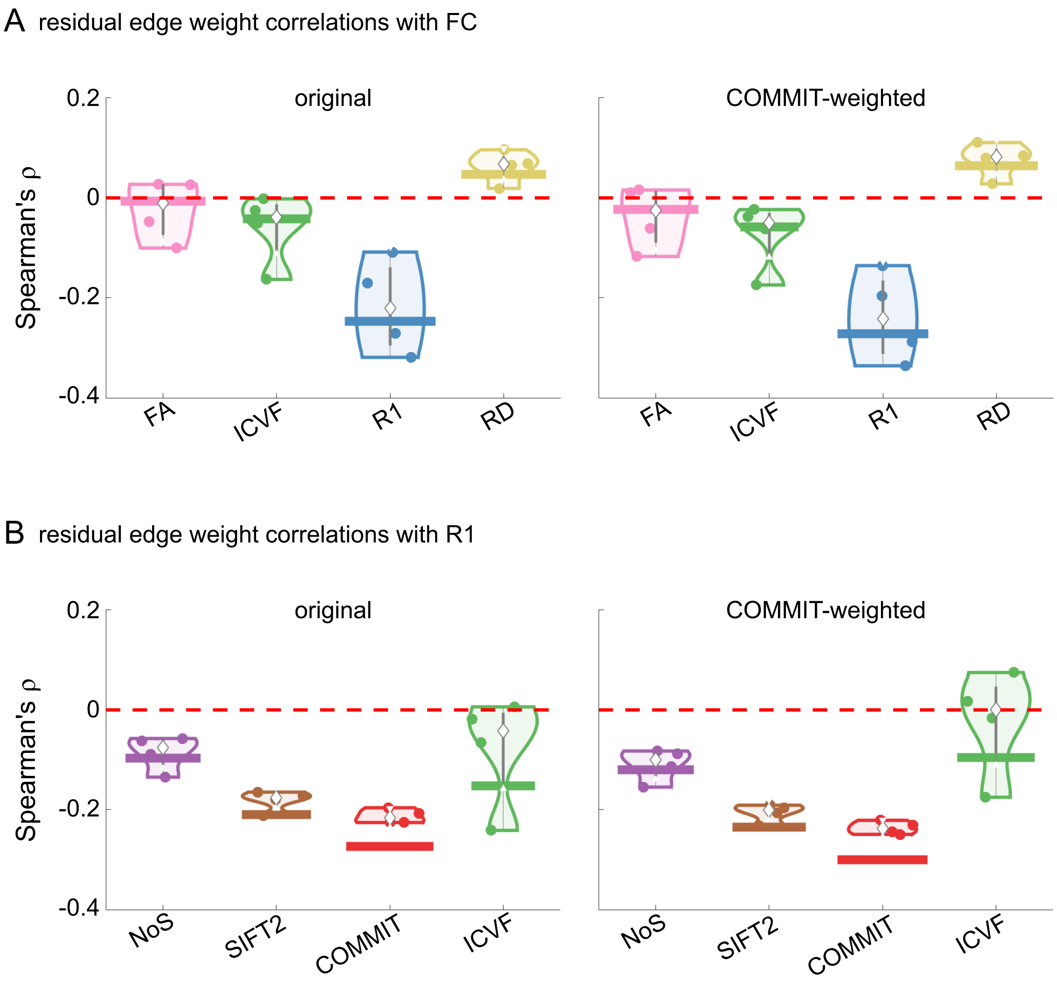

Figure S11. *Residual Edge Weight Correlations with FC and* R_1. *Edgewise Spearman's rank correlations were computed with FC (top) and* R_1 *(bottom) for networks derived using original (left) and COMMIT-weighted (right) tractometry methods. Variance due to edge length was controlled for in all networks using linear regression. Colored data points and colored bars correspond to subject-level and group-level correlation coefficients, respectively. Data from only four subjects is shown.*

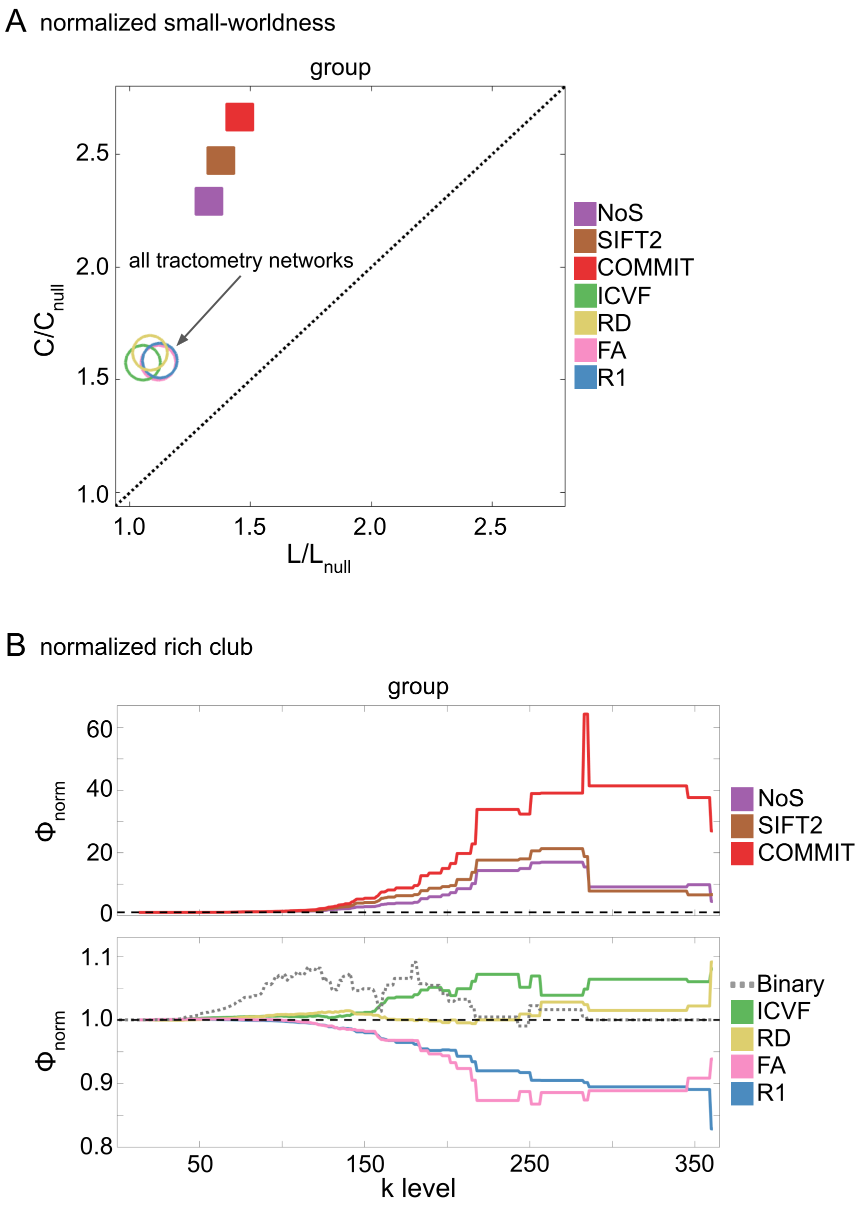

Figure S12. *Network Topology of COMMIT-Weighted Tractometry Networks. Normalized small worldness (A) and normalized rich club (B) are shown. The edge weights in tractometry networks correspond to the COMMIT-weighted average tractometry method. The normalization of all topology measures was carried out as in the main manuscript. Data from only four subjects is shown.*

Our R_1 Network is Comparable to Boshkovski et al. (2021)

To assess the comparability of our R_1-weighted SC network to that presented in Boshkovski et al. 2021, we replicated Figure 1 from their publication, which illustrates the edgewise relationship between both NoS and FA with R_1 (**Figure S13**). In an attempt to align our data more closely with theirs, we performed this analysis in the Schaefer-200 parcellation using only cortical nodes, and we did not control for edge length using linear regression.

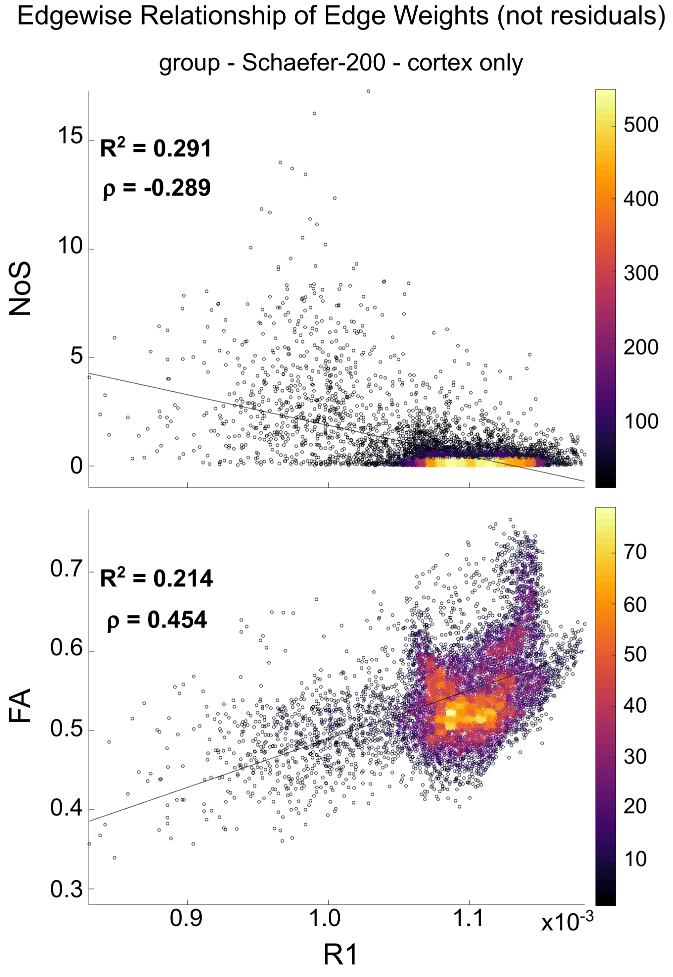

Figure S13. *Edgewise Relationship of NoS and FA with R1 Without Controlling for Edge Length.*

Here, NoS shows a negative correlation with R_1 reflected in both the negative slope of the best fit linear curve and the negative Spearman's rank correlation coefficient similar to the result we report in the main text. The magnitude of this correlation is inflated with respect to the values we report in the main text. However, that is to be expected, as we have already shown that NoS and R_1 have opposite relationships with edge length, and we did not control for this variance here. The coefficient of determination ($R^2 = 0.291$) suggests that NoS and R_1 share a portion of their variance. This contrasts with the result reported in Boshkovski et al. ($R^2 = 0.023$). This may be due to that fact that our computation of NoS networks included a step in which edge weights were scaled by the inverse of the volume of their interconnecting nodes. This normalization was not performed by Boshkovski et al.

For this reason, the relationship with FA is likely to provide a better basis of comparison between our R_1 networks. The edgewise relationship between FA and R_1 we report here appears quite similar to the result reported in Boshkovski et al. Both scatter plots (ours and theirs) suggest a positive correlation and a similar amount of shared variance between FA and R_1. Overall, our R_1 network shows a similar distribution of edge values and relationship with FA to the network reported in Boshkovski et al. Fundamental differences in our NoS networks limit comparability.
